# Activation of Interferon-Stimulated Transcriptomes and ACE2 Isoforms in Human Airway Epithelium Is Curbed by Janus Kinase Inhibitors

**DOI:** 10.21203/rs.3.rs-119695/v1

**Published:** 2020-12-11

**Authors:** Hye Kyung Lee, Olive Jung, Lothar Hennighausen

**Affiliations:** National Institute of Diabetes and Digestive and Kidney Diseases; National Institutes of Health; National Institute of Diabetes and Digestive and Kidney Diseases

**Keywords:** COVID-19, ACE2, JAK

## Abstract

SARS-CoV-2 infection of human airway epithelium activates genetic programs that lead to progressive hyperinflammation in COVID-19 patients. Here we report on genetic programs activated by interferons and the suppression by Janus kinase (JAK) inhibitors. The angiotensin-converting enzyme 2 (ACE2) is the receptor for SARS-CoV-2 and deciphering its regulation is paramount for understanding the cell tropism of SARS-CoV-2 infection. We identified candidate regulatory elements in the *ACE2* locus in human primary airway cells and lung tissue. Activating histone and promoter marks and Pol II loading characterize the intronic *dACE2* and define novel candidate enhancers distal to the genuine *ACE2* promoter and within additional introns. *dACE2,* and to a lesser extent *ACE2,* RNA levels increased in primary cells treated with interferons and this induction was mitigated by JAK inhibitors that are used therapeutically in COVID-19 patients. Our analyses provide insight into *ACE2* regulatory elements and highlight JAK inhibitors as suitable tools to suppress interferon-activated genetic programs in bronchial cells.

## Introduction

The angiotensin-converting enzyme 2 (ACE2) receptor is the gateway for SARS-CoV-2 to airway epithelium^1,2^ and the strong inflammatory response after viral infection is a hallmark in COVID-19 patients. Single cell RNA sequencing (scRNA-seq) studies have revealed that *ACE2* expression in type II pneumocytes is induced by interferons^3^, suggesting that the presence of an autoregulatory loop could result in increased viral infection. Recent studies^4,5^ have identified a novel short form of ACE2, called dACE2, that originates from an intronic promoter activated by interferons, calling into question that transcription of the full length native *ACE2* is under interferon control. Onabajo et al.^5^ used ENCODE data for chromatin modification marks (H3K4me3, H3K4me1 and H3K27ac) as well as DNase I hypersensitive (DHS) sites in cell lines to label putative regulatory elements at the newly identified exon (ex1c) located within intron 9 of the *ACE2* gene. However, no regulatory elements were detected in the vicinity of the 5’ end of the full-length transcript encoding biologically active ACE2, and in sequences distal to the genuine promoter. Since these data sets were obtained from a wide range of cell lines and not from human primary airway cells, the principal target of SARS-CoV-2, they might not present a comprehensive picture of the regulatory regions controlling expression of the *dACE2* and the full-length *ACE2* transcripts in bronchial tissue.

Janus kinase (JAK) inhibitors suppress signal transduction pathways that lead to immune activation and inflammation and they are used therapeutically to harness cytokine storm in COVID-19 patients^6,7^. However, the genetic programs activated in airway cells by interferons and their response to JAK inhibitors remain to be elucidated. In this study we pursued two linked goals. We used genome-wide chromatin profiling and RNA-seq analyses to identify the regulation of *ACE2* and *dACE2* gene expression in human primary airway cells and we investigated the ability of JAK inhibitors to blunt interferon-activated genetic programs.

## Results

### Interferons regulate the ACE2 locus in human airways cells

To comprehensively identify the genetic elements controlling the extended *ACE2* locus, with an emphasis on its interferon response, we focused on human primary Small Airway Epithelial Cells (SAEC), which express a wide range of cytokine receptors and key mechanistic components of the executing JAK/STAT signal transduction pathway (Supplementary Table 1). We stimulated SAECs with interferon type I (IFNa and IFNb), type II (IFNg) and type III (IFNl) as well as with growth hormone (GH), Interleukin 6 (IL6) and IL7, followed by RNA-seq transcriptome analyses (Supplementary Tables 2–8). Increased *ACE2* expression was obtained with the interferons but not with GH, IL6 and IL7 (Fig. 1a). However, the induction was less than that seen for classical interferon stimulated genes (ISG), such as *STAT1*, *ISG15* and *ISG20* (Fig. 1b-c). In agreement with earlier studies^4,5^, we detected the novel *dACE2* N-terminal exon (ex1c) within intron 9 of the *ACE2* gene (Fig. 1d). To obtain additional information on the interferon response of the *dACE2* and *ACE2* promoters, we used RNA-seq and determined the respective read counts over the three alternative first exons (Fig. 1d-e). While the increase of RNA-seq reads induced by IFNa/b was highest (~ 25-fold) over ex1c, a lesser, yet significant, ~ 2–10-fold increase was detected over ex1a and ex1b, supporting the notion that expression of the full-length *ACE2* transcript is also under interferon control. As an independent assay we used qRT-PCR and determined that IFN a/b stimulation led to an 8 to 15-fold increase of *dACE2* (ex1c) and an approximately ~ 3-fold increase of *ACE2* RNA (ex9) (Fig. 1f). However, the degree of induction of either form was lower than that seen for *bona fide* ISGs. Previous studies in normal human bronchial epithelium (NHBE) did not reveal an interferon response of the native *ACE2* promoter^4,5^ suggesting differences between cell types or growth conditions. The mouse *Ace2* gene is also induced by cytokines through a STAT5-based enhancer in the second intron^8^ and a DHS site is located in the equivalent location in the human *ACE2* gene in SAEC and lung tissue. This suggests the presence of additional regulatory elements controlling expression of the full-length *ACE2* mRNA.

### Interferons activate STAT enhancers in the ACE2 locus

To identify candidate regulatory elements controlling the extended *ACE2* locus, including *ACE2* and *dACE2*, in primary airway cells, we dug deeper and conducted ChIP-seq for the chromatin marks H3K27ac (activate loci), H3K4me1 (enhancers), H3K4me3 (promoters) and Polymerase II (Pol II) loading, both in the absence and presence of IFNb (Fig. 2). DNase I hypersensitive (DHS) sites from human lung tissues^9^ and SAECs^10^ served as *bona fide* predictors of regulatory regions. In addition to *ACE2*, the neighboring *TMEM27* gene, an *ACE2* homologue, as well as *BMX* are activated by interferons (Fig. 2a) suggesting the possibility of jointly used regulatory elements. *ACE2* and *TMEM27* originated from a gene duplication and their response to interferon is equivalent. The positions of the chromatin boundary factor CTCF suggests that *ACE2* and *TMEM27* are located within a sub-TAD (Fig. 2a).

In agreement with earlier studies^5^, we identified DHS sites at ex1c and in intron 17 (Fig. 2b). In addition, we identified DHS sites at ex1a, likely marking the genuine *ACE2* promoter, a distal site marking a possible enhancer and in intron 15 coinciding with activate marks. The DHS site in intron 9 overlaps with strong H3K4me3 marks, identifying it as a genuine promoter region (Fig. 2b). This site is also decorated with H3K4me1 and H3K27ac marks and extensive Pol II loading, hallmarks of a complex promoter/enhancer. H3K27ac and Pol II loading was further induced by IFNb, reflecting increased *ACE2* expression. IFNb activates the transcription factors STATs 1, 2 and 5 (Fig. 1b and Supplementary Table 3) and ChIP-seq experiments from K562 erythroid cells stimulated with IFNb^11^ revealed preferential binding of STAT5 to the intronic promoter/enhancer (Fig. 2c) further supporting regulation through the JAK/STAT pathway. The STAT1 locus served as a control for the binding of STAT transcription factors (Fig. 2d). While the *ACE2* promoter associated with ex1a is marked by a DHS site and H3K4me1 marks, there is little evidence of H3K4me3 and H3K27ac marks. However, it is well known that there is no direct relationship between gene activity and the presence of these marks.

Based on the presence of DHS sites, activating chromatin marks and Pol II loading, either in combination or by themselves, we predict additional enhancers. A prominent candidate enhancer, marked by a DHS, H3K4me1 and Pol II loading is positioned approximately 16 kb distal of ex1a and additional putative regulatory regions are located with introns (Fig. 2b). Activating histone marks and DHS sites^9,10^ are also present in the genuine *ACE2* promoter and the distal region as well as the regulatory elements of ISG, such as STAT1 and ISG15, in human lung tissues (Fig. 3).

### JAK inhibitors suppress the IFNb-sensing enhancers in the ACE2 locus

Interferons activate genetic programs through the JAK/STAT signaling pathway and JAK inhibitors are used clinically in COVID-19 patients in an effort to suppress the genomic consequences of cytokine storms^6,12,13^. To investigate if interferon-induced *ACE2* expression is controlled by the JAK/STAT pathway, we cultured SAECs in the presence of IFNb and the JAK inhibitors, Baricitinib and Ruxolitinib, followed by RNA-seq and qRT-PCR assays (Fig. 4a-c and Supplementary Tables 9–10) and ChIP-seq analyses (Fig. 4e-h). Both inhibitors suppressed the IFNb-induced increase of the full-length *ACE2* (ex1a, ex1b and ex9) and the *dACE2* (ex1c) transcripts (Fig. 4a-b), supporting that their respective promoters are under JAK/STAT control. The efficacy of the two inhibitors extended to a range of genetic programs activated through the pan JAK/STAT pathway (Figs. 4c) and induction of *bona fide* interferon stimulated genes (ISG), such as *ISG15*, was suppressed (Fig. 4d). In *ACE2*, as in other ISGs, Ruxolitinib treatment mitigated the establishment of activating H3K27ac marks and Pol II loading over distal and intronic regulatory elements (Fig. 4e-h).

Gene Set Enrichment Analysis (GSEA) of RNA-seq profiles showed, depending on the interferon, between 17% and 26% of the induced genes were linked to immune responses, like IFNg/a response, TNFa signaling, inflammatory response, IL-JAK/STAT signaling, among others (Fig. 5a and Supplementary Table 2–5). Both JAK inhibitors led to a suppression of immune signaling pathways and IFNb-induced gene activation (Fig. 5b), including both forms of *ACE2* transcripts (Fig. 4b). These data suggest that JAK inhibitors have the potential to relieve COVID-19 symptoms induced by interferon-STAT1 that contribute to the COVID-19 pathogenesis^14^.

## Discussion

SARS-CoV-2 entry into cells is facilitated by ACE2 and deciphering the regulation of the *ACE2* gene will aid our understanding of the viral tropism and also non-pulmonary pathology. Here, we have assessed the extended *ACE2* locus for regulatory elements controlling gene expression induced by interferons and other, not yet defined, stimuli. We show that the intronic regulatory region bears promoter and enhancer marks and binds STAT transcription factors, thus constituting a complex regulatory element controlling interferon-induced *dACE2* expression^5^. We also identified additional candidate regulatory elements throughout the locus that can be invoked in controlling transcription of the full-length transcripts yielding the biologically active ACE2. Regulation of the human and mouse *ACE2*^8^ loci displays distinct differences, yet they share their response to cytokines and the JAK/STAT pathway, with the mouse *Ace2* gene being activated in mammary tissue by cytokines through an intronic enhancer^8^. Further studies in tissues and primary cells are needed to better understand the complex, possibly cell-specific, regulation of the human *ACE2* locus in organs with extrapulmonary manifestations of SARS-CoV-2 infection. Our demonstration that JAK inhibitors mitigate interferon-induced activation of inflammatory programs has practical implications for their use in treating COVID-19 patients.

## Methods

### Cell culture.

Human small airway epithelial cells (SAEC) obtained from Lifeline Technology (FC-0016) were expanded using the complete BronchiaLife™ media kit (Lifeline Technology, LL-0023). All culture wares were pre-coated in 30 μγ/ml of Fibronectin (ThermoFisher Scientific, 33016015) for at least 1 h at room temperature. Calu-3 line (ATCC, HTB-55™) were cultured using Eagle’s Minimum Essential Medium (ATCC, 30–2003™) containing 10% fetal bovine serum (Cytiva, SH3007103) in 5% CO2 atmosphere at 37 °C.

Cytokines (10 ng/ml; Human IFNb, 300–02BC; Human IFNg, 300–02; Human IL6, 200–06; Human IL7, 200–07; Human Growth hormone, 100 − 40, Peprotech; Human IFNa2b, 78077.1, Stem Cell Technologies; Human IFNl3, 5259-IL-025, R&D systems) were treated in respective culture media and the cells were incubated for 12hr in 5% CO2 atmosphere at 37 °C. The cells were washed with PBS (Gibco, 14190144) twice and harvested.

Jak inhibitors, 10 μΜ of either Baricitinib (HY-15315A, MedChemExpress) or Ruxolitinib (HY-50856A, MedChemExpress), were added to BronchiLiafe™ media with or without IFNb. SAEC and incubated for 12hrs and then washed with PBS twice and harvested.

### RNA isolation and quantitative real-time PCR (qRT–PCR).

Total RNA was extracted from the collected cells and purified using the PureLink RNA Mini Kit (Invitrogen) according to the manufacturer’s instructions. cDNA was synthesized from total RNA using Superscript II (Invitrogen). Quantitative real-time PCR (qRT-PCR) was performed using TaqMan probes (ACE2, Hs01085333_m1; *STAT1*, Hs01013996_m1; *GAPDH*, Hs02786624_g1, Thermo Fisher scientific) on the CFX384 Real-Time PCR Detection System (Bio-Rad) according to the manufacturer’s instructions. Exon9 and exon1c mRNA were measured with the following primers, that were used by Onabajo et al.^5^, using SYBR Green system: Forward, 5’-GGGCGACTTCAGGATCCTTAT-3’, Reverse, 5’-GGATATGCCCCATCTCATGATGG-3’; Forward, 5’-GGAAGCAGGCTGGGACAAA-3’, Reverse, 5’-AGCTGTCAGGAAGTCGTCCATTG-3’. PCR conditions were 95 °C for 30 s, 95 °C for 15 s, and 60 °C for 30 s for 40 cycles. All reactions were done in triplicate and normalized to the housekeeping gene *GAPDH*. Relative differences in PCR results were calculated using the comparative cycle threshold (*C*_*T*_) method.

### Total RNA sequencing (Total RNA-seq) and data analysis.

Total RNA was extracted from the collected cells and purified using the PureLink RNA Mini Kit (Invitrogen) according to the manufacturer’s instructions. Ribosomal RNA was removed from 1 μg of total RNAs and cDNA was synthesized using SuperScript III (Invitrogen). Libraries for sequencing were prepared according to the manufacturer’s instructions with TruSeq Stranded Total RNA Library Prep Kit with Ribo-Zero Gold (Illumina, RS-122–2301) and paired-end sequencing was done with a HiSeq 3000 instrument (Illumina).

The raw data were subjected to QC analyses using the FastQC tool (version 0.11.9) (https://www.bioinformatics.babraham.ac.uk/projects/fastqc/). Total RNA-seq read quality control was done using Trimmomatic^15^ (version 0.36) and STAR RNA-seq^16^ (version STAR 2.5.4a) using 50 bp paired-end mode was used to align the reads (hg19). HTSeq^17^ (version 0.9.1) was to retrieve the raw counts and subsequently, R (https://www.R-project.org/), Bioconductor^18^ and DESeq2^19^ were used. Additionally, the RUVSeq^20^ package was applied to remove confounding factors. The data were pre-filtered keeping only those genes, which have at least ten reads in total. The visualization was done using dplyr (https://CRAN.R-project.org/package=dplyr) and ggplot2^21^. Genes were categorized as significantly differentially expressed with an adjusted p-value (pAdj) below 0.05 and a fold change > 2 for up-regulated genes and a fold change of < −2 for down-regulated ones. Sequence read numbers were calculated using Samtools^22^ software with sorted bam files.

### Chromatin immunoprecipitation sequencing (ChIP-seq) and data analysis.

Chromatin was fixed with formaldehyde (1% final concentration) for 15 min at room temperature, and then quenched with glycine (0.125 M final concentration). Samples were processed as previously described^23^. The following antibodies were used for ChIP-seq: H3K27ac (Abcam, ab4729), RNA polymerase II (Abcam, ab5408), H3K4me1 (Active Motif, 39297) and H3K4me3 (Millipore, 07–473). Libraries for next-generation sequencing were prepared and sequenced with a HiSeq 3000 instrument (Illumina).

The raw data were subjected to QC analyses using the FastQC tool (version 0.11.9) (https://www.bioinformatics.babraham.ac.uk/projects/fastqc/). Quality filtering and alignment of the raw reads was done using Trimmomatic^15^ (version 0.36), Bowtie^24^ (version 1.2.2) and Samtools^22^ (version 1.8), with the parameter ‘-m 1’ to keep only uniquely mapped reads, using the reference genome mm10. Picard tools (Broad Institute. Picard, http://broadinstitute.github.io/picard/. 2016) was used to remove duplicates. Homer^25^ (version 4.8.2) and DeepTools^26^ (version 3.1.3) software was applied to generate bedGraph files, separately. Integrative Genomics Viewer^27^ (version 2.5.3) was used for visualization. Each ChIP-seq experiment was conducted for more than two replicates. DeepTools was used to obtain the Pearson and Spearman correlation between the replicates.

### Statistical analysis.

For comparison of samples, data were presented as standard deviation in each group and were evaluated with a two-way ANOVA followed by Tukey’s multiple comparisons test or a one-way ANOVA with Dunnett’s multiple comparisons test using PRISM 8 GraphPad (version 8.2.0). Statistical significance was obtained by comparing the measures from wild-type or control group, and each mutant group. A value of *P < 0.05, **P < 0.001, ***P < 0.0001, ****P < 0.00001 was considered statistically significant.

## Supplementary Material

Supplement

Supplement **Supplementary Table 6.** List of all genes with normalized read counts in each replicate at Control and IL6 treated SAEC, log_2_ (fold change), *p*-value and adjusted *p*-value as well as upregulated gene list and GSEA analysis.

Supplement **Supplementary Table 1**. mRNA levels of genes associated with the pan JAK-STAT pathway in SAEC.

Supplement **Supplementary Table 10.** List of all genes with normalized read counts in each replicate at IFNb and Ruxolitinib with IFNb, treated SAEC, log_2_ (fold change), *p*-value and adjusted *p*-value as well as upregulated gene list and GSEA analysis.

Supplement

Supplement

Supplement

Supplement **Supplementary Table 8.** List of all genes with normalized read counts in each replicate at Control and GH treated SAEC, log_2_ (fold change), *p*-value and adjusted *p*-value as well as upregulated gene list and GSEA analysis.

Supplement

Supplement

Supplement

Supplement **Supplementary Table 9.** List of all genes with normalized read counts in each replicate at IFNb and Baricitinib with IFNb, treated SAEC, log_2_ (fold change), *p*-value and adjusted *p*-value as well as upregulated gene list and GSEA analysis.

Supplement **Supplementary Table 7.** List of all genes with normalized read counts in each replicate at Control and IL7 treated SAEC, log_2_ (fold change), *p*-value and adjusted *p*-value as well as upregulated gene list and GSEA analysis.

Supplement **Supplementary Table 5.** List of all genes with normalized read counts in each replicate at Control and IFNl3 treated SAEC, log_2_ (fold change), *p*-value and adjusted *p*-value as well as upregulated gene list and GSEA analysis.

Supplement

Supplement

Supplement

Supplement **Supplementary Table 3.** List of all genes with normalized read counts in each replicate at Control and IFNb treated SAEC, log_2_ (fold change), *p*-value and adjusted *p*-value as well as upregulated gene list and GSEA analysis.

Supplement

Supplement

Supplement **Supplementary Table 2.** List of all genes with normalized read counts in each replicate at Control and IFNa treated SAEC, log_2_ (fold change), *p*-value and adjusted *p*-value as well as upregulated gene list and GSEA analysis.

Supplement

Supplement **Supplementary Table 4.** List of all genes with normalized read counts in each replicate at Control and IFNg treated SAEC, log_2_ (fold change), *p*-value and adjusted *p*-value as well as upregulated gene list and GSEA analysis.

Supplement

Supplement

Supplement

Supplement

Supplement

Supplement

Supplement

Supplement

Supplement

Supplement

Supplement

Supplement

Supplement

Supplement

Supplement

Supplement

Supplement

## Data Availability

All data were obtained or uploaded to Gene Expression Omnibus (GEO). ChIP-seq for STATs was obtained under GSE31477. ChIP-seq data for H3K27ac and H3K4me1 from human lung tissues were downloaded from GSE143115 and 142958. DNase I hypersensitive (DHS) data from human lung tissues and SAEC were obtained under GSE90364 and 29692, respectively. The RNA-seq data and ChIP-seq for SAECs in the presence of cytokines and the JAK inhibitors were uploaded to GSE161665 (ChIP-seq in GSE161663 and RNA-seq in GSE161664).
